# A Rare Case of Bruck Syndrome Type 2 in Siblings With Broad Phenotypic Variability

**DOI:** 10.31486/toj.18.0145

**Published:** 2020

**Authors:** Lindsey Luce, Michael Casale, Sean Waldron

**Affiliations:** ^1^ Department of Orthopedic Surgery, Medical University of South Carolina, Charleston, SC; ^2^ Department of Orthopedic Surgery, Ochsner Clinic Foundation, New Orleans, LA; ^3^ The University of Queensland Faculty of Medicine, Ochsner Clinical School, New Orleans, LA

**Keywords:** *Bruck syndrome 2*, *scoliosis*, *pediatrics*

## Abstract

**Background:** Bruck syndrome is a rare autosomal recessive condition that presents with many of the symptoms of osteogenesis imperfecta. In addition to defective type I collagen, manifesting as bone fragility, osteoporosis, and blue sclera, Bruck syndrome is additionally characterized by arthrogryposis with pterygia. Joint contractures are frequently bilateral and severe.

**Case Report:** We report the medical record and radiographic data for 2 siblings with Bruck syndrome type 2—a male (age 6 years) and a female (age 5 years)—born to nonaffected parents. The male has experienced more than 45 fractures, developed severe scoliosis, and has debilitating flexion contractures. The female has minimal flexion contractures, a history of 15 fractures, and severe scoliosis.

**Conclusion:** The dramatic difference between the phenotypes of these 2 cases is significant because it is the largest known variability of phenotypic presentation in siblings. Previous cases of siblings with differing presentations at birth have been reported, but the extent of these differences is not as extreme as in our cases. Because Bruck syndrome presents similarly to osteogenesis imperfecta and could be clinically mistaken for a form of osteogenesis imperfecta if contractures are minimal, a reasonable focus for research efforts is the development of genetic diagnostic protocols for osteogenesis imperfecta with the goal of ruling out Bruck syndrome.

## INTRODUCTION

Osteogenesis imperfecta is a primarily autosomal dominant disorder involving defective type I collagen that manifests as bone fragility, osteoporosis, blue sclera, and hearing loss.^1^ Bruck syndrome is a rare collagen disorder that presents with all of these symptoms of osteogenesis imperfecta, excluding hearing loss, and is additionally characterized by arthrogryposis with pterygia.^1,2^ Joint contractures are frequently bilateral and severe.^2^

Bruck syndrome occurs in multiple types, with the distinguishing factor being the collagen gene in which a mutation has occurred. Bruck syndrome types 1 and 2 are the most common forms, involving mutations of the FKBP10 and PLOD2 collagen genes, respectively.^1,3^ There are no known phenotypic differences between types 1 and 2.^1,4^ Inheritance patterns for all types of Bruck syndrome have been described as autosomal recessive.^2,5^ Bruck syndrome, like osteogenesis imperfecta, is not known to be associated with any language or cognitive developmental milestone delays. We present the cases of 2 siblings who both have Bruck syndrome but who presented with markedly different phenotypic pictures.

## CASE REPORT

The mother of the 2 children has no known collagen disorder or other genetic condition. The father also has no known collagen disorder; however, he has a mildly abnormally shaped skull with a questionable history of premature sagittal suture closure in infancy.

### Male Sibling

The older child is a 6-year-old male who presented at birth with generalized hypotonia, plagiocephaly, flexion contractures of the knees and ankles, left talipes equinovarus, right vertical talus, torticollis, multiple rib fractures, and a left clavicle fracture. He was born via caesarean section because of breech position. He was born preterm at 36 weeks, but the pregnancy was otherwise uncomplicated. He has met all language and cognitive developmental milestones to date. Genotyping revealed a mutation of the PLOD2 gene, consistent with Bruck syndrome type 2.

**Figure 1.**
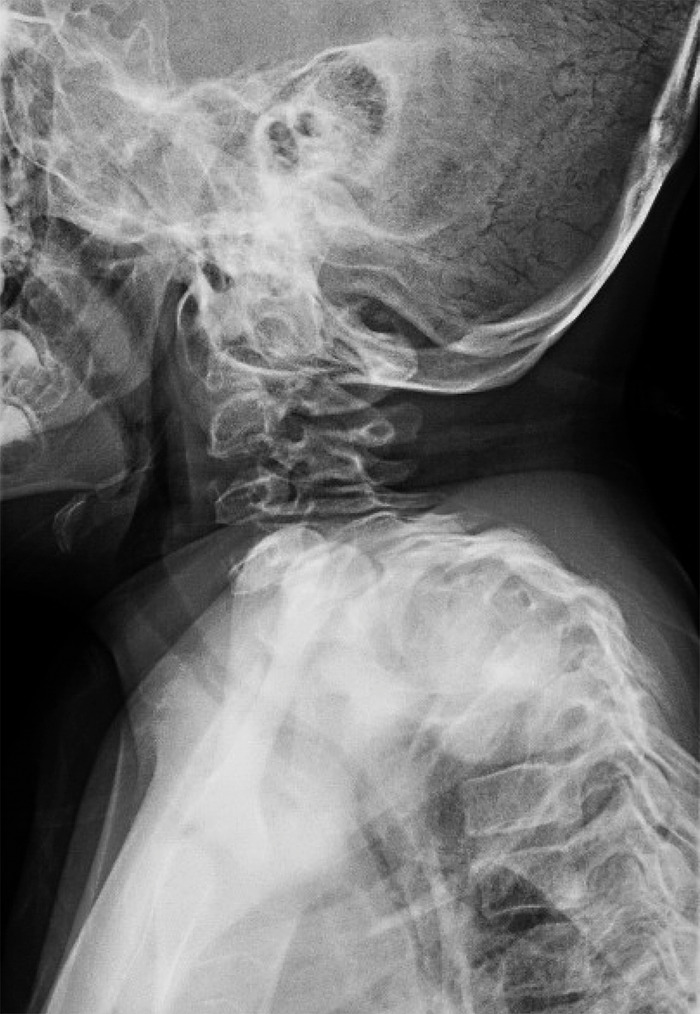
**Radiograph of cervical and thoracic spine of the male sibling at age 4 showing severe spinal deformity.**

**Figure 2.**
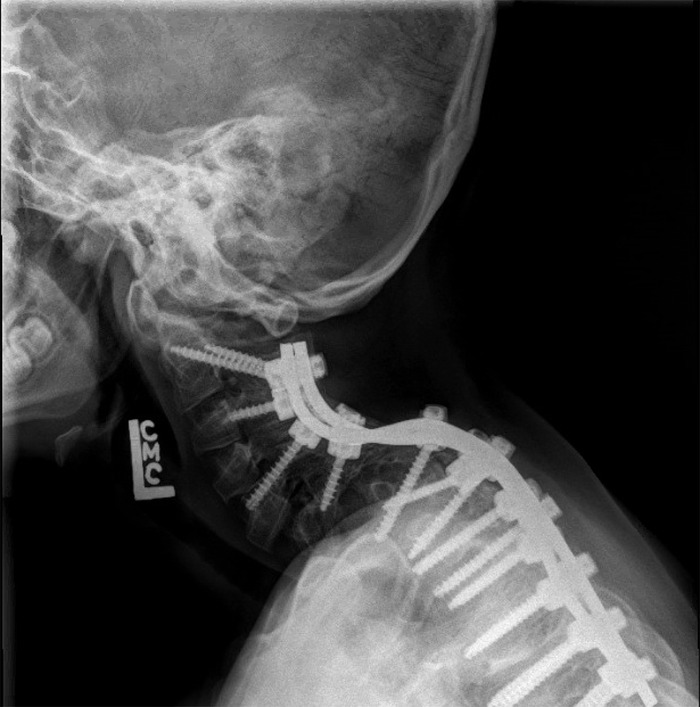
**Lateral radiograph of cervical spine of the male sibling after undergoing posterior spinal fusion for scoliosis at age 5.**

He has generalized upper extremity hypotonia with poor truncal control. His skull has normalized since birth, but he remains mildly deformed with plagiocephaly and frontal bossing. He underwent bilateral serial casting followed by serial knee-ankle-foot orthotic (KAFO), bracing for attempted correction of his knee and ankle flexion deformity from age 3 months until 2 years of age. Repeated bilateral Achilles and hamstring tenotomies have also been performed. Despite this management, he continues to have severe bilateral knee and ankle joint contractures. By age 3, he developed severe cervicothoracic scoliosis (Figure 1) consisting of a complex segmentation anomaly from the C6 to the T1 vertebra, resulting in severe levoscoliosis of the cervicothoracic spine. He underwent posterior fusion of C2 to T6 in 2014 at the age of 5 (Figure 2).

**Figure 3.**
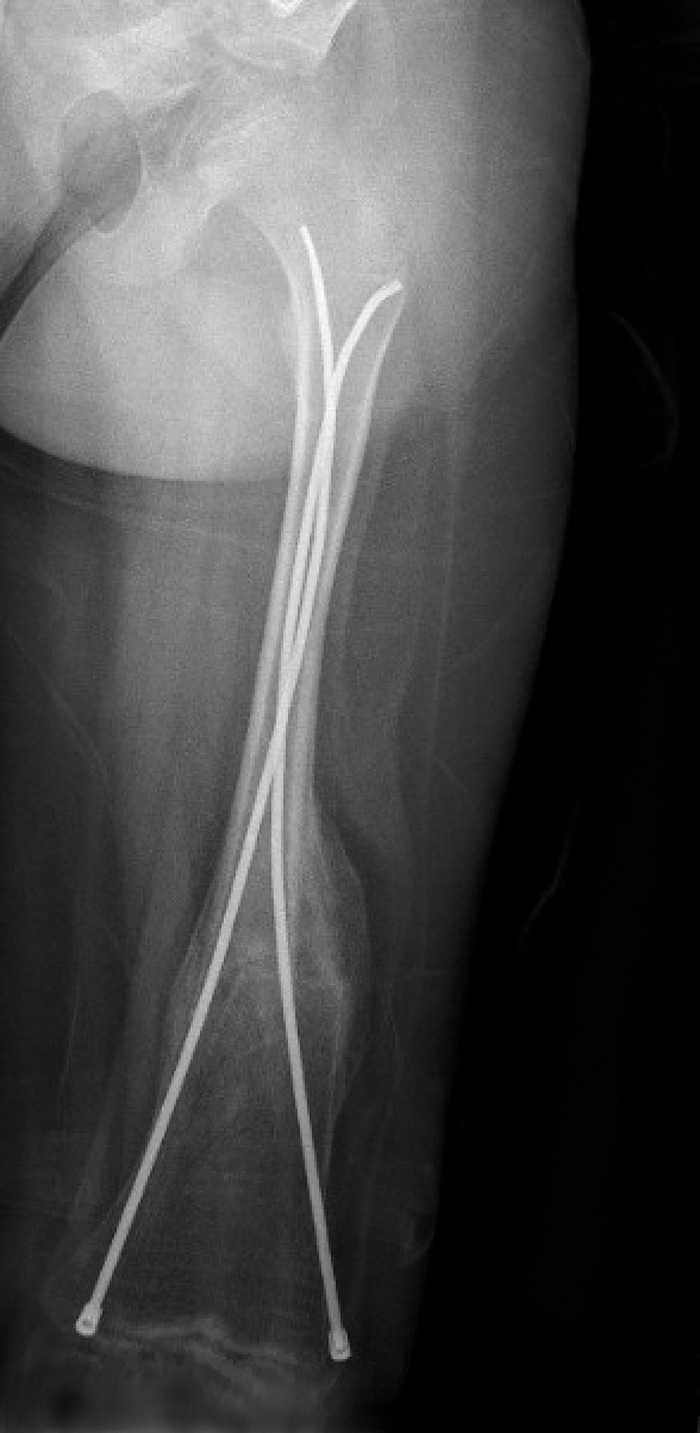
**Radiograph of left femur of the male sibling showing a healed femur fracture after intramedullary nailing at age 4.**

To date, he has a history of more than 45 fractures, with many requiring either intramedullary nailing or open reduction with internal fixation. His left femur has been the most frequently affected by the condition, with at least 5 fractures and significant deformity occurring as a result (Figure 3). Because of this deformity and severe joint contractures affecting his bilateral hips, knees, and ankles, the child is unable to walk and only able to weight bear for short periods of time. His treatment plan includes the implantation of a telescoping nail to correct the deformity of his left femur.

His spine bone mineral density (BMD) at age 6 is 0.459 g/cm^2^, giving him an age-matched Z-score of –2.3. He has consistently been measured at less than the first percentile for height with respect to age and sex since turning 5 years old. In addition to the aforementioned treatments, he has been managed with physical therapy for serial stretching to increase joint mobility, regular pamidronate infusions, and vitamin D and calcium supplementation. At present, he requires full-time care and assistance and will continue to do so in the future.

### Female Sibling

The younger child is a 5-year-old female who presented at birth with frontal bossing, micrognathia, a low-set right ear, and multiple rib fractures. She had no contractures or extremity fractures at birth. She was born via caesarean section at term after an uncomplicated pregnancy. She has met all language and cognitive milestones to date. PLOD2 gene testing revealed the same mutation as her brother, giving her a diagnosis of Bruck syndrome type 2.

**Figure 4.**
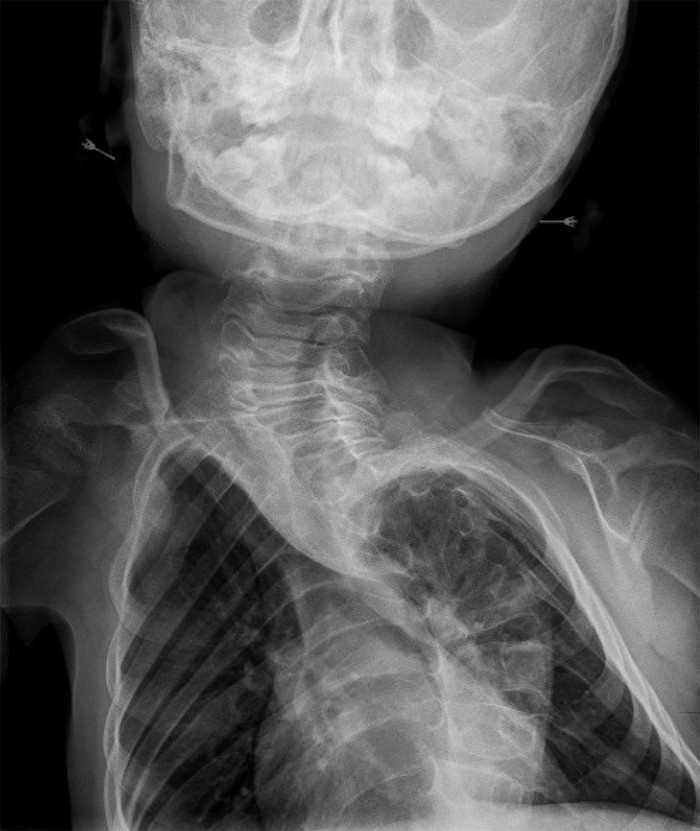
**Radiograph of cervical and thoracic spine of the female sibling at age 5 showing severe spinal deformity and autofusion.**

**Figure 5.**
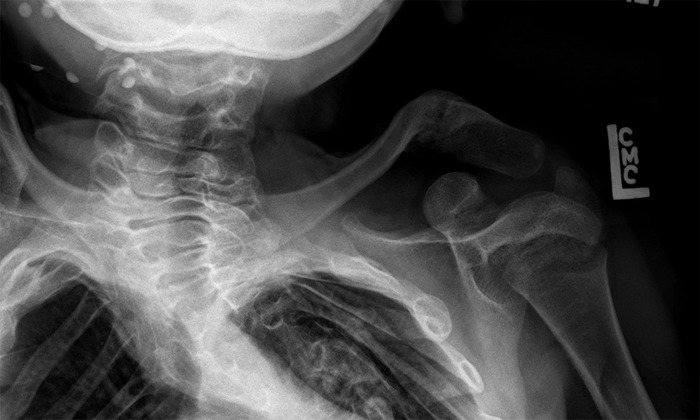
**Radiograph of the left shoulder girdle of the female sibling at age 5 showing abnormally shaped and hypoplastic clavicle, scapula, and proximal humerus.**

She has persistent midface hypoplasia and micrognathia. She has developed severe thoracic levoscoliosis with anterior wedging of the T7 and T10 vertebrae and autofusion in her thoracic spine that was first noticeable at age 4 (Figure 4). Her scoliosis is progressively worsening with growth; she has yet to undergo correction. She also has congenital malformation of the left shoulder consisting of an abnormally shaped and hypoplastic clavicle, scapula, and proximal humerus (Figure 5).

To date, she has had approximately 15 known fractures, none of which have required operative treatment. She has minimal lower extremity joint contractures and is able to walk with a walker, although walking is becoming more difficult with the progression of her scoliosis.

Her spine BMD at age 5 is 0.342 g/cm^2^, giving her an age-matched Z-score of –3.1. In comparison to her brother (less than first percentile after age 5), her short stature is less severe, measuring at the first to fifth percentile for height with respect to age and sex. To date, her medical treatment has also consisted of physical therapy for serial stretching, regular pamidronate infusions, and vitamin D and calcium supplementations. Ultimately, this patient will require spinal fusion surgery to correct her scoliosis and hopefully maintain her ability to ambulate.

## DISCUSSION

The phenotypic differences between the 2 siblings are summarized in the Table. Phenotypic variability has been widely evident in Bruck syndrome, most notably in reports of siblings with the disease.^6,7^ Variability appears to exist with respect to the extent of bone fragility, as well as both the severity and prevalence of contractures. Multiple joint contractures have been the defining characteristic of Bruck syndrome since its first description in 1897.^2,8^ Arthrogryposis remains the primary characteristic of Bruck syndrome that distinguishes it from osteogenesis imperfecta.

**Table.** Summary of Phenotypic Differences Between the Siblings

	Male, Age 6 years		Female, Age 5 years	
Phenotype	Description	Age of Onset/Progression	Description	Age of Onset/Progression
Contractures	Severe flexion contractures of knee and ankle joints, bilaterally	Birth/Persistent but less severe after multiple tenotomies	No lower extremity contractures at birth	N/A
	No upper extremity joint contractures	N/A	No upper extremity joint contractures	N/A
Fractures	More than 45 fractures	Left clavicle and multiple rib fractures at birth/Persistent in frequency; many open reductions required	Approximately 15 fractures	Rib fractures present at birth/No open reductions required
Spinal deformities	Severe cervicothoracic scoliosis, complex segmentation anomaly C6-T1	Severe status by age 3/ Spinal fusion at age 5	Moderate thoracic scoliosis, wedging of T7 and T10	Severe status by age 4/ Yet to undergo correction
Skull and facial deformities	Plagiocephaly, frontal bossing, bilateral proptosis, wormian bones	Birth/Normalized to mild deformity since birth	Midface hypoplasia, micrognathia	Birth/Persistent
Height for age, sex	Less than first percentile	Age 6 years	Between first and fifth percentiles	Age 5 years
Bone mineral density	0.459 g/cm^2^Age-matched Z-score of –2.3	Age 6 years	0.342 g/cm^2^Age-matched Z-score of –3.1	Age 5 years
Weight-bearing status	Pulls to stand, unable to walk	Persistent	Pulls to stand, walks with aid	Ability to walk decreasing with progression of scoliosis
Other	Left talipes equinovarus, right vertical talus	Birth/Only moderately improved despite serial KAFO bracing, Achilles tenotomies	No foot/ankle deformity	N/A
	No shoulder/upper extremity deformity	N/A	Misshapen and small clavicle, scapula, and proximal humerus	Birth

KAFO, knee-ankle-foot orthotic; N/A, not applicable.

Bruck syndrome tends to have a worse prognosis than osteogenesis imperfecta because the multiple joint contractures complicate functional ability and fracture healing. In patients with cerebral palsy, multiple joint contractures have been well documented to increase the fracture rate and to complicate fracture healing.^9^ Patients with a more severe contracture phenotype have decreased functional capacity at baseline, such as in the case of the male child. In contrast to his younger sister, he has been managed with multiple lower extremity tenotomies, yet he is unable to walk because of the postoperative recurrence of contractures and fracture-induced deformities. The younger sister has had far fewer fractures and less severe short stature than her brother despite her lower spine BMD.

The dramatic difference between the phenotypes of these 2 cases is significant in that it represents the largest variability of phenotypic presentation in siblings that is known to the authors. Previous cases of siblings having differing presentations at birth, tendencies toward fracture, severities of contractures, and functional capacities have been reported; however, the extent of these differences is not as extreme as in the siblings presented here.^6^

Given the rarity of Bruck syndrome, no major efforts to develop treatment options for the disease have been undertaken that are distinct from the efforts for osteogenesis imperfecta. The current nonoperative treatment modalities are nearly identical to those used for osteogenesis imperfecta and consist of regular bisphosphonate infusions and vitamin supplementation. These treatments have been proven to be effective for both disease processes; however, the prognosis for Bruck syndrome is more severe given the complications of joint contractures.^10^

When joint contractures are not pronounced, distinguishing between Bruck syndrome and osteogenesis imperfecta without genotyping may be difficult, as the fracture patterns on radiographic imaging and the physical examination may be congruous. Both autosomal recessive and autosomal dominant inheritance patterns have been described for osteogenesis imperfecta, and distinguishing Bruck syndrome from the rare autosomal recessive subtype of osteogenesis imperfecta may be difficult, as multiple loci on the FKBP10 and PLOD2 genes have been implicated in both disease processes.^1,3,5^ Thus, more specific genetic analysis may be necessary to delineate between these diseases if the existence of joint contractures is minimal.

Because Bruck syndrome presents similarly to osteogenesis imperfecta and could be clinically mistaken for a form of osteogenesis imperfecta if contractures are minimal, a reasonable focus for research efforts is genetic diagnostic protocols for osteogenesis imperfecta with the goal of ruling out Bruck syndrome. Such protocol development would be beneficial for families and physicians as early genetic analysis and pedigree assessment are essential for accurate diagnosis and prognostication for individuals and the future offspring of affected families.

## CONCLUSION

These siblings demonstrate dramatic differences in phenotypes representative of Bruck syndrome. Future research aimed at distinguishing between osteogenesis imperfecta and Bruck syndrome at the genetic level will be key for diagnostic and prognostic purposes, as the presence of severe joint contractures drastically changes the treatment and outcomes of patients with Bruck syndrome compared to patients with osteogenesis imperfecta alone.

## Notes

This article meets the Accreditation Council for Graduate Medical Education and the American Board of Medical Specialties Maintenance of Certification competencies for Patient Care and Medical Knowledge.
